# Identification of novel drought-responsive miRNA regulatory network of drought stress response in common vetch (*Vicia sativa*)

**DOI:** 10.1515/biol-2021-0109

**Published:** 2021-10-11

**Authors:** Yongqun Zhu, Qiuxu Liu, Wenzhi Xu, Li Yao, Xie Wang, Hong Wang, Yalin Xu, Linxiang Li, Chunhua Duan, Zhixin Yi, Chaowen Lin

**Affiliations:** Soil and Fertilizer Research Institute, Sichuan Academy of Agricultural Sciences, Chengdu, Sichuan 610066, People’s Republic of China; Bazhong Green Agriculture Innovation and Development Research Institute, Sichuan Academy of Agricultural Sciences, Bazhong, Sichuan 636000, People’s Republic of China

**Keywords:** common vetch, *Vicia sativa*, microRNA, drought stress, high-throughput sequencing, target gene

## Abstract

Drought is among the most important natural disasters with severe effects on animals and plants. MicroRNAs are a class of noncoding RNAs that play a crucial role in plant growth, development, and response to stress factors, including drought. However, the microRNAs in drought responses in common vetch (*Vicia sativa*), an annual herbaceous leguminous plant commonly used for forage by including it in mixed seeding during winter and spring, have not been characterized. To explore the microRNAs’ response to drought in common vetch, we sequenced 10 small RNA (sRNA) libraries by the next-generation sequencing technology. We obtained 379 known miRNAs belonging to 38 families and 47 novel miRNAs. The two groups had varying numbers of differentially expressed miRNAs: 85 in the comparison group D5 vs C5 and 38 in the comparison group D3 vs C3. Combined analysis of mRNA and miRNA in the same samples under drought treatment identified 318 different target genes of 123 miRNAs. Functional annotation of the target genes revealed that the miRNAs regulate drought-responsive genes, such as leucine-rich repeat receptor-like kinase-encoding genes (LRR-RLKs), ABC transporter G family member 1 (ABCG1), and MAG2-interacting protein 2 (MIP2). The genes were involved in various pathways, including cell wall biosynthesis, reactive oxygen removal, and protein transport. The findings in this study provide new insights into the miRNA-mediated regulatory networks of drought stress response in common vetch.

## 1 Introduction

Common vetch is an annual high-yielding leguminous forage with high crude protein content and good palatability and is mainly used for hay, silage, and green manure [1,2]. It is an irreplaceable component in mixed cropping because of its high yields, forage quality, and capacity to increase soil nitrogen and promote the build up of organic matter content [3,4]. These attributes, in turn, promote soil fertility, which reduces fertilizer application, thereby reducing environmental pollution and production costs [5]. Besides, common vetch is low in fat, has a high starch content, and its protein content is about twice that of many grains, [6] and hence, suitable as a low-cost substitute for animal feed [6]. Compared to other forages, common vetch has the best usage potential index when used singly as a ruminant feed [7] and improves the nutritional quality of mixed fodder. Under cultivation, common vetch is more tolerant to extreme weather conditions such as winter and drought than other cultivated food legumes [8] and perennial forage legumes [9]. For example, common vetch can withstand up to 24 days of drought, and its biological functions are fully restored upon watering [10].

Common vetch, being a drought-tolerant forage, can be grown in marginal zones, which presents great potential as a sustainable forage source [11]. Drought is a common environmental stress that negatively affects plant growth leading to excessive production of reactive oxygen species (ROS) [12]. To attenuate the effects of ROS, the antioxidant levels in plants are increased as a plant tolerance mechanism against drought and other abiotic stresses. For example, manganese superoxide dismutase (Mn-SOD) is expressed in rice [13], and there is overexpression of ascorbic peroxidase (APX) in tobacco chloroplasts under drought stress [14]. Similarly, there is overexpression of monodehydroascorbate reductase (MDAR) in transgenic tobacco under salt stress [14], while the overexpression of dehydroascorbic acid reductase (DHAR) enhances plant tolerance against various abiotic stresses [15]. This suggests that genes encoding antioxidant enzymes improve plant abiotic stress tolerance, including drought tolerance.

Drought stress inevitably damages the plant cell membranes [16]. The membrane system of plants creates a barrier between cells and the external environment, which is crucial for maintaining the microenvironment and normal metabolism of the cells. Membrane phospholipids are sensitive to ROS damage, which causes peroxidation of membrane lipids [17], leading to Malondialdehyde (MDA). The accumulation of MDA causes electrolytes to leak out of the cells, resulting in increased conductivity. Thus, increased electrical conductivity (EC) can be used as a marker of cell membrane damage and drought resistance in plants [18].

MicroRNAs (miRNAs) are small noncoding RNAs of 18–24nt in length. They negatively regulate the expression of downstream target genes by degrading or inhibiting translation [19,20,21], which plays important regulatory roles at various stages of plant growth [22]. They play critical regulatory roles in enhancing plant adaptability to stress conditions by modulating plant response to drought stress [23,24,25,26,27,28]. For example, miR156 expression can promote flowering under drought stress, which mitigates the damaging effects of drought stress [29]. Similarly, the expression level of miR160 in transgenic tobacco is downregulated under drought stress, thereby enhancing its drought tolerance [30]. Moreover, the expression levels of some conserved miRNAs, such as miR159, miR167, miR169, and miR397, are up- or downregulated under drought or high salt stress conditions [25,30,31].

Therefore, the objective of this study was to identify the novel and known miRNAs expressed in common vetch under drought stress and analyze miRNA-mediated regulatory networks in drought stress response. The findings in this study will enhance the understanding of molecular coordination mechanisms in response to drought stress and promote the genetic improvement of drought tolerance in common vetch.

## 2 Materials and methods

### 2.1 Treatments and sampling

The common vetch cultivar “Chuanbei,” a breeding variety well adapted to the southwest region of China [32], was used in the present study. Common vetch seeds were sterilized using 75% ethanol for 5 min and then rinsed with sterile distilled water. The seeds (10 g per pot) were planted in sterilized quartz in plastic pots (20 cm in length, 15 cm width, and 8 cm deep). The pots were kept in a controlled growth chamber at the Sichuan Agricultural University in Chengdu. The chamber had a 12 h photoperiod, 19°C/15°C day/night temperature, and 75% relative humidity. Thirty-five days after planting, the plants were incubated with 25% (w/v) polyethylene glycol (PEG) 6000 dissolved in Hoagland’s solution for five days to induce drought stress. Control plants were treated with Hoagland’s solution without PEG. The aboveground parts of the control plants were sampled at 0 days (C0), day 3 (C3), and day 5 (C5), and at day 3 (D3) and day 5 (D5) in drought-stressed plants and immediately stored in liquid nitrogen. One plant per treatment was sampled. There were two biological replicates per treatment.

### 2.2 RNA isolation, library construction, and sequencing

Total RNA was extracted from the aboveground part of plant samples using the Trizol reagent (TransGen, China) following the manufacturer’s instructions. sRNA libraries were prepared using 3 μg of the total RNA as the input material. Sequencing libraries were generated using the NEBNext® Multiplex Small RNA Library Prep Set for Illumina® (NEB, USA) following the manufacturer’s instructions. Subsequent clustering of the index-coded samples was then performed on a cBot Cluster Generation System using a TruSeq SR Cluster Kit v3-cBot-HS (Illumia) according to the manufacturer’s instructions.

### 2.3 Reads mapping, alignment of known miRNAs, and prediction of novel miRNAs

The sRNA tags without mismatches were mapped to the reference sequences using Bowtie (http://bowtie.cbcb.umd.edu) [33] to analyze their expression and distribution. Mapped sRNA tags were then used to identify known miRNA. miRBase20.0 was used as the reference database, while the modified mirdeep2 software [34] and srna-tools-cli were used to determine the potential miRNAs and draw their secondary structures. Custom scripts were used to obtain the miRNA counts and base bias on the first position of the identified miRNA with various lengths and on each position of all identified miRNAs. Hairpin structure characteristics of miRNA precursors were used to predict novel miRNA. MiREvo [35] and mirdeep2 [34] software were employed to predict the novel miRNAs.

### 2.4 Differentially expressed miRNAs in response to drought stress

Differential expression analysis for two conditions was performed using the DESeq R package (version 1.8.3). The gene ontology (GO) function was used for enrichment analysis of the candidate target gene of differentially expressed miRNAs. After that, the KOBAS software was used to identify the significantly enriched candidate target gene in the KEGG pathways [36].

### 2.5 RT-qPCR validation of miRNA sequencing data

RT-qPCR was used to validate the accuracy of the miRNA sequencing data. Total RNA was isolated from the drought-stressed and control plants using Trizol reagent (TransGen, China). The first-strand cDNA for RT-qPCR analysis was synthesized from 1,000 ng of total RNA using HiScript™ Reverse Transcriptase (Vazyme, Nanjing, China) according to the manufacturer’s instructions. Six miRNAs were selected for validation. The primers were designed using Prime5 software (Table S1). The RT-qPCR reaction was performed using Bio-Rad CFX96 following the manufacturer’s instructions with Taq Pro Universal SYBR qPCR Master Mix(#Q712) (Vazyme Biotech Co., Ltd). The PCR reaction was carried out in a total volume of 20 μL containing 1 μL of cDNA, 0.5 μL of forward primers, 0.5 μL of reverse primers, 10 μL Taq Master Mix solution, and 8 μL of RNase-free water. All the RT-qPCR reactions were conducted in three replicates. The relative expression levels were quantified using the 2^−ΔΔt^ method [37].

## 3 Results

### 3.1 Overview of transcriptome dynamics and sRNA sequencing

Ten sRNA libraries were constructed to explore the molecular mechanisms of drought response in common vetch. A total of 134,222,486 raw reads were generated. The datasets generated were deposited in the National Center for Biotechnology Information database [BioProject PRJNA681445]. Subsequently, the reads were filtered and cleaned to remove the low-quality reads, those containing ploy-N, 5′ adapter contaminants, ploy A, T, G, and C, and those without 3′ adapter or the insert tag, obtaining 129,577,792 clean reads (Table 1).

**Table 1:** Summary of sRNA libraries of common vetch under drought stress

Library	Raw reads	N% > 10%	Low-quality reads	5′ Adapter contamine	3′ dapter null	Ploy A/T/G/C	Clean reads
C0-1	15,399,906	59 (0.00%)	65,251 (0.42%)	6,661 (0.04%)	1,755,349 (11.40%)	6,255 (0.04%)	13,566,331 (88.09%)
C0-2	12,703,957	16 (0.00%)	13,199 (0.10%)	7,217 (0.06%)	176,218 (1.39%)	31,978 (0.25%)	12,475,329 (98.20%)
C3-1	16,623,813	15 (0.00%)	16,677 (0.10%)	11,214 (0.07%)	199,285 (1.20%)	27,765 (0.17%)	16,368,857 (98.47%)
C3-2	12,510,514	9 (0.00%)	11,121 (0.09%)	42,384 (0.34%)	327,115 (2.61%)	20,770 (0.17%)	12,109,115 (96.79%)
C5-1	13,292,233	343 (0.00%)	40,572 (0.31%)	6,785 (0.05%)	237,217 (1.78%)	33,776 (0.25%)	12,973,540 (97.60%)
C5-2	11,802,347	315 (0.00%)	36,975 (0.31%)	5,657 (0.05%)	211,620 (1.79%)	32,381 (0.27%)	11,515,399 (97.57%)
D3-1	13,164,735	328 (0.00%)	36,593 (0.28%)	13,411 (0.10%)	333,750 (2.54%)	19,667 (0.15%)	12,760,986 (96.93%)
D3-2	12,213,823	318 (0.00%)	34,747 (0.28%)	7,334 (0.06%)	216,747 (1.77%)	20,372 (0.17%)	11,934,305 (97.71%)
D5-1	12,870,298	324 (0.00%)	37,365 (0.29%)	5,316 (0.04%)	229,422 (1.78%)	20,103 (0.16%)	12,577,768 (97.73%)
D5-2	13,640,860	322 (0.00%)	39,353 (0.29%)	5,912 (0.04%)	278,851 (2.04%)	20,260 (0.15%)	13,296,162 (97.47%)
Total	134,222,486						129,577,792 (96.54%)

Note: (1) N: Undetermined base information (2) N% > 10%: The proportion of filtered reads with undetermined base information in the total number of raw reads exceeding 10%. (3) 5′ Adapter Contamine: The proportion of filtered reads with 5′ adapter contamine in the total raw reads. (4) 3′ Adapter Null: The proportion of filtered reads without 3′ joints in the total raw reads. (5) PloyA/T/G/C: The proportion of filtered reads due to the presence of ployA/T/G/C in the total raw reads.

**Figure 1:**
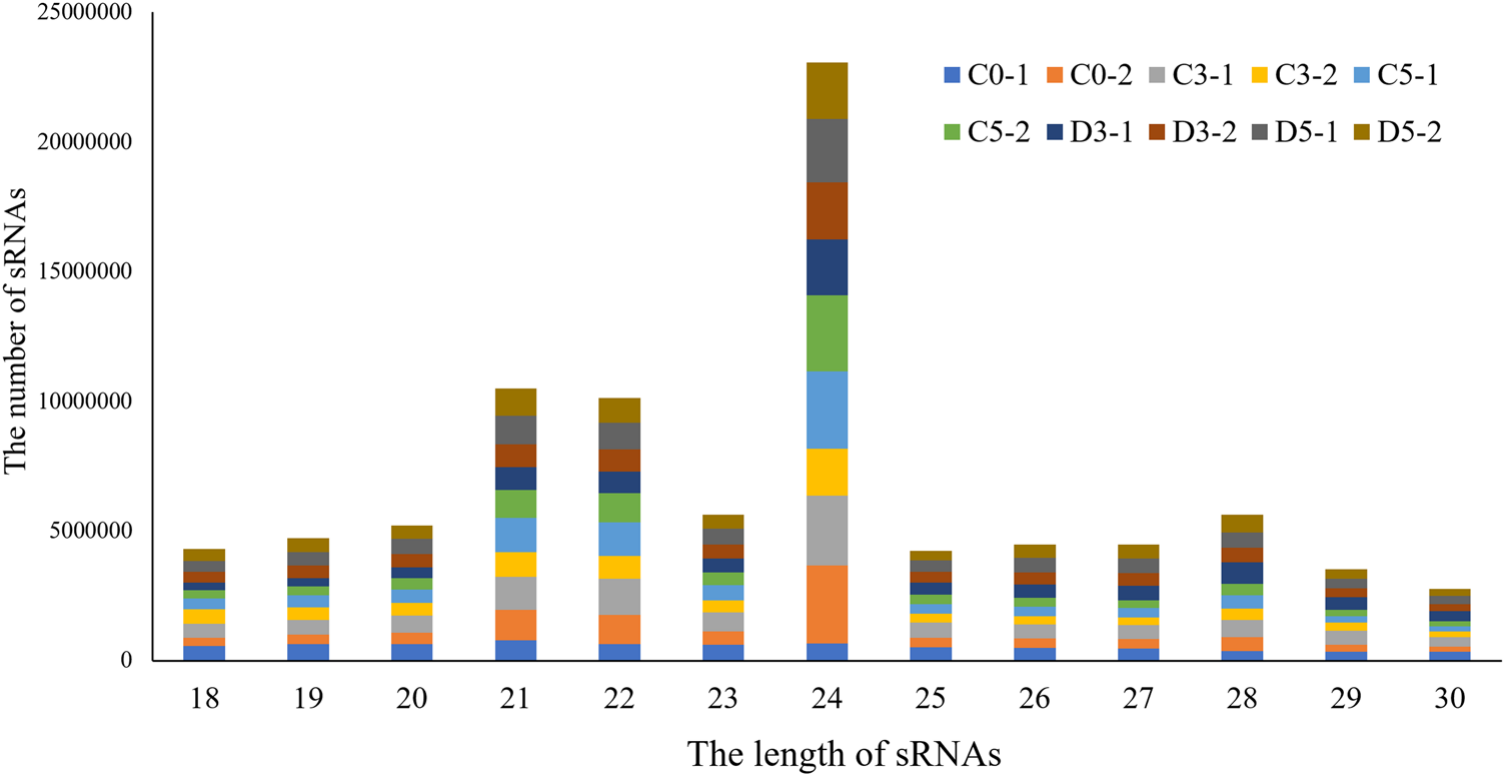
Small RNAs length distribution in common vetch. The *X*-axis shows the length of sRNAs, while the *Y*-axis shows the number of sRNAs identified. Different colors denote the ten libraries.

Clean reads of 24nt (26.01%) in length accounted for the largest proportion of clean reads, while reads of 21–24nt collectively accounted for 55.60% of all the reads (Figure 1). The 24nt sRNAs represent small interfering RNAs (siRNAs) directly involved in RNA-directed DNA methylation, while the 24nt/21nt ratio indicates the degree of DNA methylation [38,39]. The results revealed that the methylation levels of both the control and drought-stressed plants increased over time (Figure 1).

The unique sequences were compared with known plant miRNA sequences in the miRBase database to identify the conserved known miRNAs. The sRNAs were assigned into eight categories based on a BLASTN search and further sequence analyses: known miRNAs, rRNA, tRNA, snRNA, snoRNA, novel miRNA, TAS, and unannotated sRNAs. Each library had an average of 1,174 known miRNAs and 398 novel miRNAs (Table 2). Moreover, a significant proportion of unique sequences (>90% in all treatments) had not previously been identified and annotated, suggesting that common vetch has many novel miRNAs.

**Table 2:** Distribution of small RNAs among the different categories in common vetch

Library	Total	Known miRNA	rRNA	tRNA	snRNA	snoRNA	Novel miRNA	TAS	Unannotated
C0-1	633,999	637	39,376	2	1,087	1,263	111	183	591,340
	100%	0.10%	6.21%	0.00%	0.17%	0.20%	0.02%	0.03%	93.27%
C0-2	1,021,425	1,324	34,727	2	1,095	1,347	497	1,599	980,834
	100.00%	0.13%	3.40%	0.00%	0.11%	0.13%	0.05%	0.16%	96.03%
C3-1	1,088,582	1,281	45,534	1	1,943	1,926	480	1,197	1,036,220
	100.00%	0.12%	4.18%	0.00%	0.18%	0.18%	0.04%	0.11%	95.19%
C3-2	880,605	1,335	43,458	1	1,737	1,935	469	1,268	830,402
	100.00%	0.15%	4.94%	0.00%	0.20%	0.22%	0.05%	0.14%	94.30%
C5-1	1,159,086	1,348	37,018	4	1,489	1,694	567	1,530	1,115,436
	100.00%	0.12%	3.19%	0.00%	0.13%	0.15%	0.05%	0.13%	96.23%
C5-2	1,069,208	1,326	34,267	1	1,303	1,560	494	1,466	1,028,791
	100.00%	0.12%	3.20%	0.00%	0.12%	0.15%	0.05%	0.14%	96.22%
D3-1	848,088	1,092	32,569	1	1,774	2,298	317	1,061	808,976
	100.00%	0.13%	3.84%	0.00%	0.21%	0.27%	0.04%	0.13%	95.39%
D3-2	843,771	1,114	33,452	1	1,560	1,576	332	1,213	804,523
	100.00%	0.13%	3.96%	0.00%	0.18%	0.19%	0.04%	0.14%	95.35%
D5-1	963,398	1,130	38,485	3	1,696	1,899	350	1,291	918,544
	100.00%	0.12%	3.99%	0.00%	0.18%	0.20%	0.04%	0.13%	95.34%
D5-2	949,441	1,152	37,309	1	1,783	1,870	365	1,530	905,431
	100.00%	0.12%	3.93%	0.00%	0.19%	0.20%	0.04%	0.16%	95.36%

Note: (1) rRNA/tRNA/snRNA/snoRNA: Refers to the proportion of rRNA/tRNA/snRNA/snoRNA in each library. (2) Novel miRNA: Indicates the proportion of new miRNAs identified in each library. (3) TAS: Refers to the proportion of TAS gene in each library. (4) Unannotated: Indicates proportion of sRNAs in each library that were not successfully annotated.

### 3.2 Known and novel miRNAs in drought-stressed common vetch

**Figure 2:**
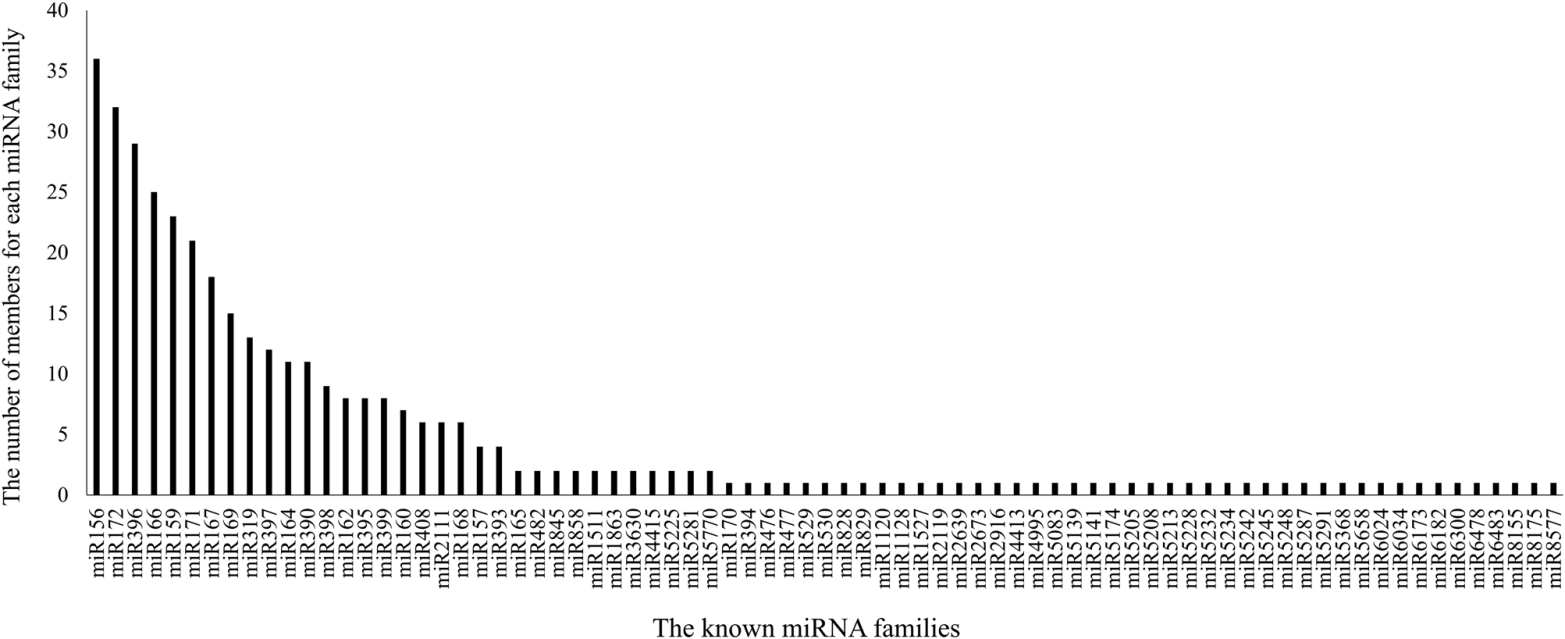
Distribution of known miRNA families in common vetch. *Y*-axis represents the number of members in each miRNA family. The *X*-axis represents the known miRNA families.

A total of 379 unique sequences belonging to 78 known miRNA families were identified in all libraries (Figure 2 and Table S2). The largest miRNA family was miR156 with 36 members, followed by miR172 and miR396, respectively.

There were significant differences in abundance among the miRNA families, as shown in Table S2. The average transcripts per million reads (TPM) for each miRNA in ten libraries were calculated to compare their expression levels [40,41,42]. After TPM standardization (Table S3), the highest relative expression level obtained was mtr-miR5213-5p (243058.37), followed by ath-miR159a (94704.81) and gma-miR319d (74525.87), respectively.

Prediction of novel miRNAs identified 47 novel miRNAs (Table S4), all of which were 20nt to 25nt in length. The majority of the novel miRNAs were 21nt (53.19%) in length. Expression analysis further revealed that the expression levels of the 47 miRNAs were below 10 read counts except for novel_2 *, novel_119*, novel_17*, novel_131*, novel_60*, and novel_54* (Table S5). Base distribution results for each position in the miRNAs revealed that uracil (U) occupied the first position in the novel miRNA present in the 10 libraries (Figure A1).

### 3.3 Differentially expressed miRNAs in drought-stressed common vetch

**Figure 3:**
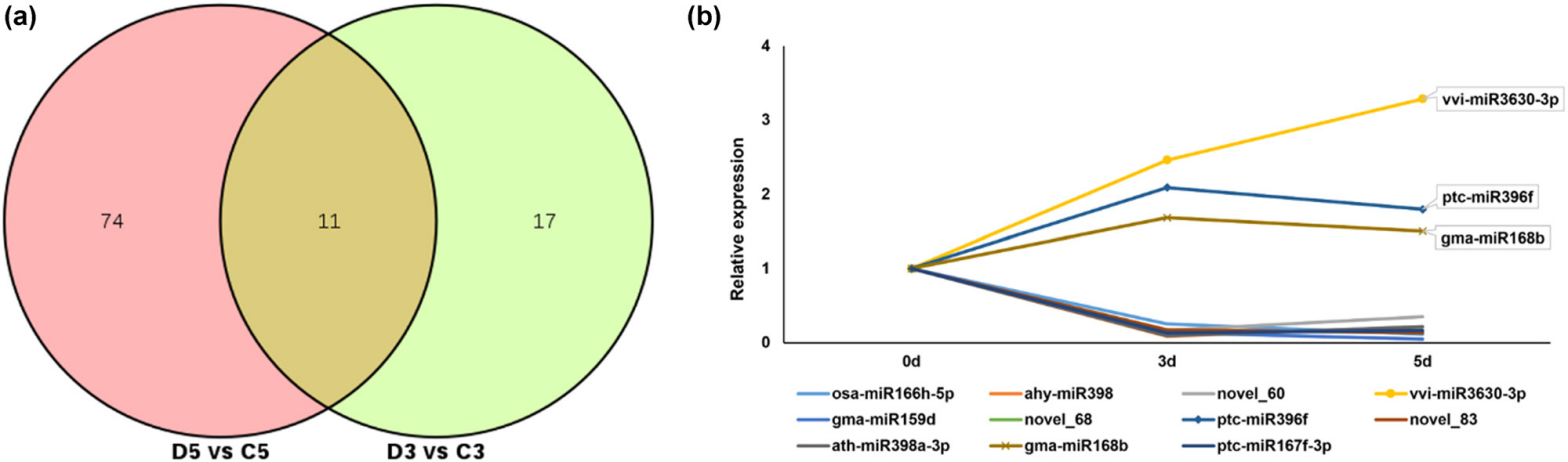
Differential expression analysis of miRNAs in common vetch under drought stress. (a) Venn diagram of (D5 vs C5) vs (D3 vs C3). (b) The relative expression levels of the 11 common genes in the two comparison groups.

**Figure 4:**
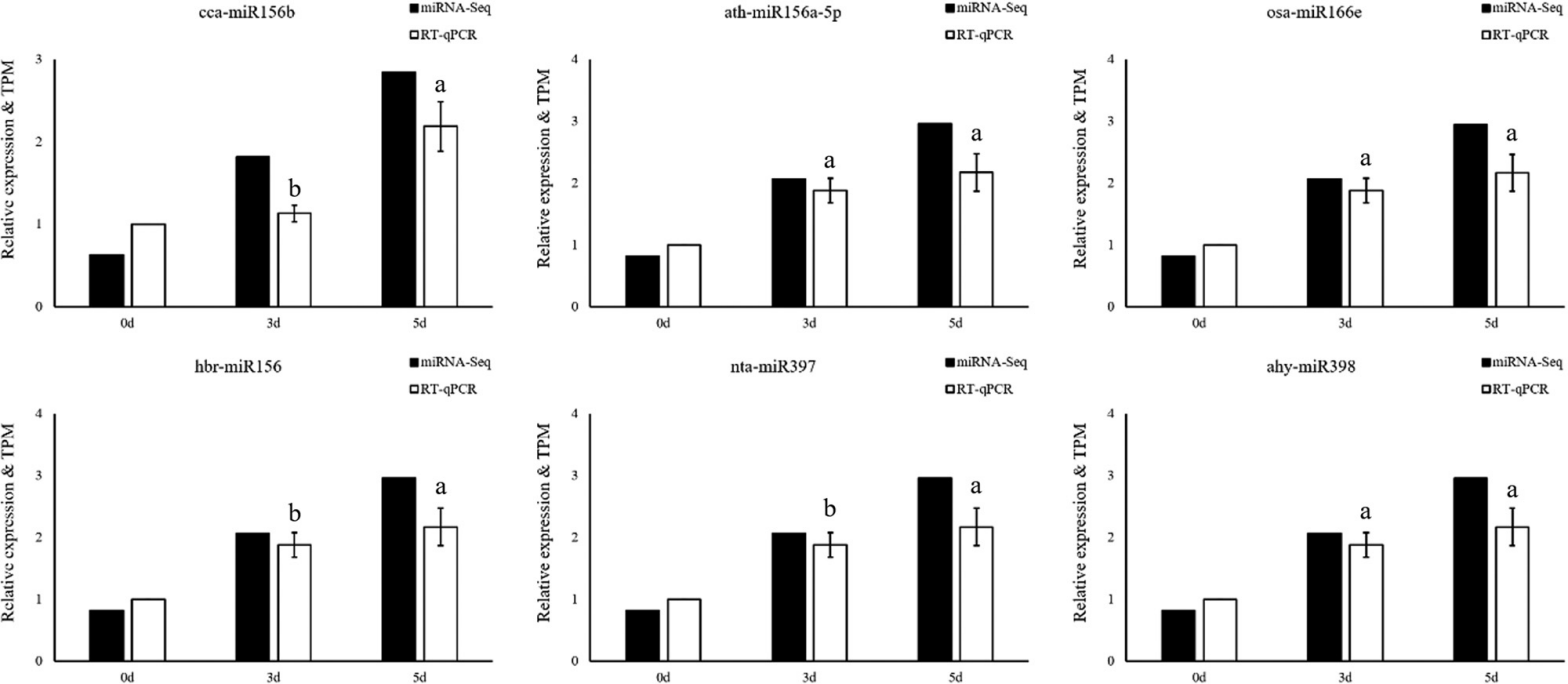
RT-qPCR validation of differentially expressed miRNAs under drought stress in common vetch.

The DESeq was used for differential expression analysis of miRNAs in each sample. In total, 85 differentially expressed miRNAs (37 upregulated and 48 downregulated) in D5 vs C5 and 28 differentially expressed miRNAs (9 upregulated and 19 downregulated) in D3 vs C3 (Figure 3a) were identified. The Venn diagram of (D5 vs C5) vs (D3 vs C3) further revealed 11 common genes in the two comparisons, including ahy-miR398, gma-miR159d, osa-miR166h-5p, among others. These results indicate that the expression of these 11 genes constantly changes during drought stress. Further analysis of the relative expression levels of the 11 genes revealed that the expression of vvi-miR3630-3p, ptc-miR167f-3p, and gma-miR168b increased under drought stress, while the other genes were downregulated (Figure 3b). To confirm the miRNA-Seq data, selected miRNAs that showed differential expression of miRNA under stress were chosen. The RT-qPCR validation revealed that the differentially expressed miRNAs had a similar expression pattern to the sequencing data (Figure 4). This implies that the data produced in this study are reliable.

### 3.4 Prediction and annotation of target genes for drought-responsive miRNAs

**Figure 5:**
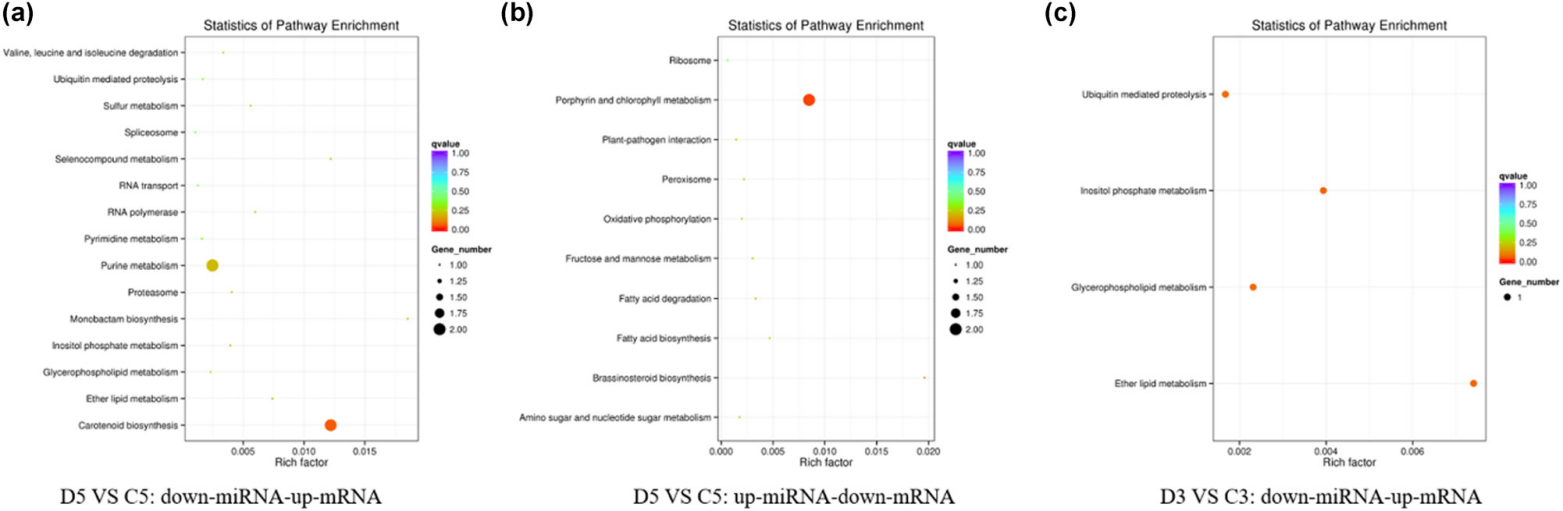
KEGG enrichment analysis of differentially expressed target genes. (a), (b) and (c) respectively represented the comparison group D5 vs C5：down-miRNA-up-mRNA，D5 vs C5：down-mRNA-up-miRNA and D3 vs C3：down-miRNA-up-mRNA.

GO and KEGG pathway analysis of the target genes of differentially expressed miRNAs was done to explain further the change of miRNA in common vetch under drought stress. A total of 283 differentially expressed target genes were predicted in 85 differentially expressed miRNAs in the comparison group D5 vs C5. Gene ontology enrichment analysis revealed that most of the differentially expressed target genes were related to metal ion binding, cation binding, and transition metal ion binding. The KEGG enrichment analysis revealed that the genes were significantly enriched in the nitrogen metabolism pathway. The different target genes corresponding to different-miRNA were classified based on their differential expression profiles (up-miRNA-down-mRNA and down-miRNA-up-mRNA). Two genes annotated as abscisic acid 8′-hydroxylase (played a key role in abscisic acid biosynthesis) in the carotenoid biosynthesis were found to be significantly enriched in the down-miRNA-up-mRNA group (Figure 5a). In the up-miRNA-down-mRNA group, clusters 8152.47925 and 8152.47927 in porphyrin and chlorophyll metabolism were significantly enriched (Figure 5b). The genes were thus annotated as divinyl chlorophyllide, an 8-vinyl-reductase that regulates the synthesis of protochlorophyllide. In addition, ath-miR156a-5p was significantly upregulated. However, its predicted target genes cluster 8152.61131 and cluster 8152.8716 were downregulated. Cluster 8152.61131 was annotated as PCX1: a member of the LRR-RLK family, which regulates cell wall construction [43]. MicroRNA ath-miR156a was significantly upregulated and its predicted target gene cluster 8152.40191 was involved in anthocyanin synthesis. Cluster 8152.79559 was annotated as a driver protein involved in cell wall synthesis. Besides, cluster 8152.84378 was involved in protein ubiquitination, which strongly indicated it would respond to drought stress from multiple vantage points.

In the comparison group D3 vs C3, 35 differentially expressed target genes were predicted in the 28 differentially expressed miRNAs. Only 16 pairs of differentially expressed target genes and miRNAs were present in the down-miRNA-up-mRNA group. KEGG enrichment analysis revealed that cluster 8152.56666 was the most significantly enriched and was annotated as phospholipase C. Meanwhile, cluster 8152.56666 was enriched in ether fat metabolism, inositol phosphate metabolism, and glycerophospholipid metabolism pathways (Figure 5c). Further analysis revealed that novel_68* had varying degrees of downregulated expression in the two comparison groups (FC_D5 vs C5_ = −2.3147 and FC_D3 vs C3_ = −1.9144). Its target gene was cluster 8152.40257, annotated as ABCG1 and related to peroxidase and ATP synthesis. ABCG1 plays a vital role in the detoxification of plant ROS [44].

## 4 Discussion

### 4.1 miRNA profiling of drought stress in common vetch

MicroRNAs are a class of small noncoding RNAs that play a crucial role in gene regulation. Numerous studies postulate that miRNAs widely respond to environmental stress by regulating gene expression [45,46,47,48,49,50,51]. For example, during drought stress, different miRNAs are expressed in *Arabidopsis thaliana* [23], rice [52], maize [53], and wheat [54]. In *A. thaliana*, several miRNAs such as miR396, miR168, and miR398 are upregulated under drought conditions [23]. Similarly, several miRNAs, such as miR159, miR1871, and miR397, are regulated in rice under drought stress [52]. A similar phenomenon has been observed in maize subjected to drought conditions where some miRNAs (miR156, miR159, miR160, and miR398) are upregulated while others, such as miR319, miR396, and miR399, are downregulated [53]. In wheat, miR156, miR159, and miR168 are upregulated, while miR171, miR395, and miR916 are downregulated under drought stress [44]. Most of the miRNAs that regulate plant response to drought stress are evolutionarily conserved across different plant species. Herein, many sRNA sequencing data were obtained in common vetch seedlings under drought stress. For example, miR396 was upregulated under drought stress in common vetch and *Arabidopsis thaliana* [55], but was downregulated by 37% in pitaya fruit under drought stress [56]. This difference may be due to the different expression levels of miR396 at different time scales in response to drought stress in the plant. While in pitaya, the downregulation of miR396 reached a minimum level (−37%) after 20 h [56], in common vetch, miR396 reached its highest level after 5 days, which was an upregulation by about two times. miR396 is a growth-regulating factor (GRF) [57,58], which causes different response degrees when plants are stressed at different growth stages.

Moreover, some drought-responsive miRNAs in common vetch regulate other biological and abiotic stresses. For example, miRNA169 and miRNA319 play key roles in response to abscisic acid, salt stress, and drought stress [48,59,60]. Similarly, miRNA398 is considered a bridge connecting plant responses to oxidative stress and other stresses (water deficit, salt stress, ABA, and biological stress) [61]. This is because these miRNAs partially regulate a gene that responds to different environmental stresses. Nonetheless, the specific molecular mechanism of drought-responsive miRNAs identified herein should be verified in future studies.

### 4.2 miRNA-mediated regulatory network of drought stress

**Figure 6:**
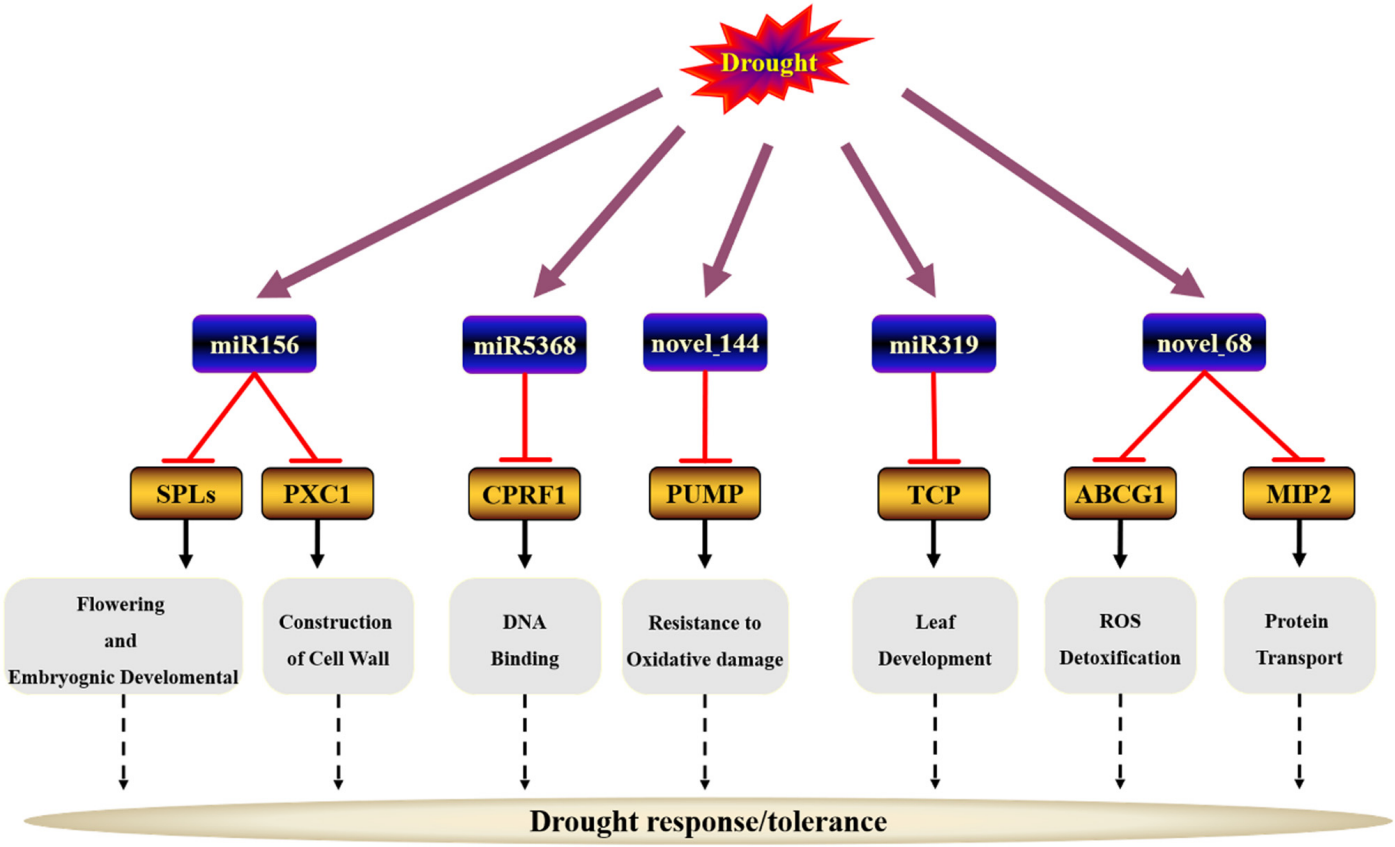
The proposed miRNA-based model for regulating plant drought response in common vetch.

MicroRNAs regulate gene expression by up- or downregulating target genes. Numerous studies have revealed the expression patterns of miRNAs and their target genes under plant drought stress through high-throughput sequencing [52,62]. Herein, a large data set of miRNAs was obtained. The data set was jointly analyzed with mRNA data to identify specific drought-responsive genes. Based on those results, a miRNA-based model for regulating plant drought response was proposed (Figure 6).

## 5 Conclusion

This study identified and characterized 379 known miRNAs belonging to 38 families and 47 novel miRNAs using sRNA sequencing and bioinformatics approaches. In total, 85 miRNAs were differentially expressed in the comparison group D5 vs C5 and 38 in D3 vs C3 under drought stress. Furthermore, functional annotation of the target genes provided further evidence for their possible involvement in drought stress. The findings in this study contribute valuable information for future studies on the function of miRNAs in common vetch and other plants during drought stress.
